# Effect of Zn/Mg Ratios on Microstructure and Stress Corrosion Cracking of 7005 Alloy

**DOI:** 10.3390/ma12020285

**Published:** 2019-01-16

**Authors:** Shuai Wang, Binghui Luo, Zhenhai Bai, Chuan He, Sizhi Tan, Gen Jiang

**Affiliations:** Key Laboratory for Nonferrous Materials (MOE), School of Materials Science and Engineering, Central South University, Changsha 410083, China; 163101020@csu.edu.cn (S.W.); zhenhaibai@yeah.net (Z.B.); hechuan@csu.edu.cn (C.H.); tsz_csu@csu.edu.cn (S.T.); jianggen2017@csu.edu.cn (G.J.)

**Keywords:** 7005 aluminum alloy, peak-aged state, phase transformation, resistance of stress corrosion cracking

## Abstract

The effects of different Zn/Mg ratios on the microstructure, mechanical properties and resistance of stress corrosion cracking of peak-aged 7005 aluminum alloy were investigated. It was found that the Zn/Mg ratio played a very important role in controlling the aging time, the electrical conductivity of the hardness peak point and the resistance of stress corrosion cracking of the alloy. With the increase of Zn/Mg ratio (wt. %), the time taken by the alloy to achieve the peak hardness value gradually increases aging at 120 °C. When the Zn/Mg ratio is in the range from 2.27% to 2.62%, the precipitate phase of the alloy after peak-aged is mainly dominated by smaller disc-like η’ phase and GP I (Guinier Preston) zones, the grain boundary precipitates are slender and continuous and the PFZ (precipitate free zones) is narrow. However when this value is in the range from 3.01% to 4.08%, precipitation phase in matrix of the alloy is mainly composed of short-rod η′ phase and GP II zones, the precipitation phases within the grain boundary are large and distribute intermittently and the PFZ is narrower. The results of SSRT (slow strain rate tests) show that when Zn/Mg ≥ 3.61, the 7005 aluminum alloy at peak-aged has good resistance of stress corrosion cracking in 3.5% NaCl + 0.5% H_2_O_2_ aqueous solution. However, when Zn/Mg ≤ 3.01, the strength of the alloy sharply decreases in 3.5% (wt. %) NaCl + 0.5% (wt. %) H_2_O_2_ aqueous solution.

## 1. Introduction

7005 aluminum alloy is widely used in rail transportation and aerospace industry due to its light weight, high strength, good deformability, and good weldability [1,2,3]. As a key load-bearing component, 7005 aluminum alloy is required to have good resistance of stress corrosion cracking (SCC) under certain special corrosive environments. According to the failure analysis of the test data of 7005 aluminum alloy in high-speed trains in recent years, 7005 aluminum alloy often breaks under some lower stress, and most of the failures occur in the corrosive environment where Cl^−^ and H^+^ coexist. Existing literature studies have shown that the poor resistance of 7xxx aluminum alloy series to SCC has been overcame using it in over-aged state (T7), which reduces its yield strength about 10–15% comparing to the T6 condition. In terms of the heat treatment process, such as the retrogression and re-aging (RRA), low-temperature pre-aging (LPA), and non-isothermal aging (NIA), have been developed. However, these above mentioned heat treatments are not only complicated, but it decreases the part of the yield strength of the alloy [4,5,6,7,8]. Both of the composition and the heat treatment system are key factors affecting the mechanical strength and stress corrosion resistance of the alloy [9,10,11,12]. Therefore, it is very meaningful to study the relation between the alloy composition and the microstructure of the alloy in the peak-aged (T6) state. As known the elemental ratio of the alloy not only determines the type, quantity, and distribution of the precipitate phase, but more importantly, the elements segregation along the grain boundary plays an important role in stress corrosion cracking and tensile behavior [13,14,15,16,17,18]. The elemental ratio is often used to study the relation between elemental composition and equilibrium phase, but is rarely used to study the composition, size and distribution of the metastable phase. The common metastable phase of 7xxx ally contain GP zones, η’ phase and η_p_. The usual precipitation sequence of 7xxx series Al alloys can be summarized as: Solid solution → Guinier Preston zones (GPZs) → Metastable η’ phase → Stable η’ phase (MgZn_2_). GPZs and Metastable η’ phase is believed to be responsible for peak hardening of 7xxx series Al alloys. Therefore, in order to obtain the best performance, proper control of early precipitation, especially the size and density of these precipitates is crucial. It has also been generally acknowledged that two types of GP zones may form in 7xxx series Al alloys [19,20,21,22,23]. GP I zones are reported to form as ordered and coherent layers of Zn and Mg/Al atoms on {100}_Al_ and these ones generally exhibit spherical morphology [4]. GP I zones are easily observed by bright field transmission electron microscopy (BFTEM). Probably due to its incomplete internal order [21], GP I zones have not been observed by high resolution transmission electron microscopy (HRTEM). These precipitates form from 25 °C up to about 140 °C and they serve as the precursor of the η’ phase and η phase [21]. GP II zones are believed as Zn-rich layers on {111}_Al_ planes and formed after quenching from temperatures above 450 °C and aging at temperatures above 70 °C [19,23]. GP II zones also can be served as the precursor of the η’ phase and η phase. Positron annihilation spectroscopy (PAS) investigations indicate that the interaction between solute atoms and vacancies, especially Mg-vacancy, Zn-vacancy, or Zn_2_-vacancy complex, have a significant effect on GP zone formation [19,20,21]. The mall angle X-ray scattering (SAXS) experiment [24] has suggested that the coexistence of two types of particles with different sizes aged between about 60 °C and 100 °C in 7xxx series Al alloys, one of which is smaller GP zones and the other is larger η’ particles. In this study, the Zn and Mg ratios were used to study the microstructure changes of 7005 alloy under the peak-aged state. In addition the influence of Zn/Mg ratio on the stress corrosion resistance of the alloy was discussed.

## 2. Materials and Methods

The composition elements of 7005 aluminum alloy contain Zn (4.20–4.95 wt. %), Mg (1.20–1.90 wt. %), Mn (0.20–0.7 wt. %), Cu (≤0.20 wt. %), Cr, Zr (≤0.30 wt. %), and the balance is Al. The pre-designed Zn contents were 4.20 wt. %, 4.60 wt. %, and 4.95 wt. % respectively; the Mg contents were 1.20 wt.%, 1.60 wt. %, and 1.90 wt. % respectively; the other element content including Mn, Cu, Cr, and Zr is 0.25 wt. %, 0.10 wt. %, 0.15 wt. % and 0.15 wt. %, respectively. The highest, middle and lowest limits of Zn and Mg were combined to obtain five alloys including Zn/Mg ratios of 2, 2.5, 3, 3.6 and 4, respectively. These alloys were cast by gravity casting method following by smelting, homogenization at 475 °C, and the alloy plates were rolled by 15% percentage reduction per pass into thick sheet about 2–3 mm with 90% deformation or so. After hot rolling the alloy sheets were cut into sample pieces of 10 mm × 10 mm × 2 mm in dimensions to test hardness. The samples were solution treated at 475 °C for 1.5 h and then quenched in water at room temperature (25 °C). The series of as-quenched samples were then immediately aged at 120 °C. As shown in Table 1, ICP-AES test was used to confirm the element contents of alloys. Inductively coupled plasma atomic emission spectrometry (ICP-AES, SPECTRO BLUE SOP, Spectro, Kleve, Germany) is a spectral analysis method using an inductively coupled plasma moment as an excitation source. It has high accuracy and precision, low detection limit, fast measurement, wide linear range, and simultaneous determination of various elements and other advantages. The hardness was tested using the HV-5 Vickers hardness tester (HV-5HV-5, ShuangXun, Shanghai, China) with a load in 0.5 kg and holding for 15 s. The conductivity test was indicated by the D60K digital metal eddy current conductivity meter (MM.1-D60K, Beijing, China) and the conductivity at the coordinate of 0 hwas used to indicate the as-quenched state. The microstructure of these samples was observed by using the FEI Tecnai G2 20ST transmission electron microscope (TEM, FEI, Hillsboro, OR, USA) operating at 200 kV. Fracture morphologies was observed by using the scanning electron microscopy (SEM, Quanta-200, FEI, OR, USA). The corresponding tensile samples were taken along the T-L direction of the sheet, and slow strain rate test was performed. The resistance of stress corrosion cracking can be assessed by the ratio of the mechanical properties in the corrosive medium to that in the inert medium. The smaller the value, the more the corrosion sensitive it is with respect to a particular corrosive environment. In this experiment, the resistance of stress corrosion cracking (Rscc) of 7005 aluminum alloy in 3.5% NaCl + 0.5% H_2_O_2_ aqueous solution was evaluated by the following formula:

R_SCC_ = σ_y_(NaCl + H_2_O_2_)/σ_y_(air)

and the slow strain rate was 8.34 × 10^−7^ s^−1^. σ_y_ indicates the yield strength of the materials. 

## 3. Results

### 3.1. Hardness and Conductivity in Relation with the Zn/Mg Ratio of the Alloy 

**Figure 1:**
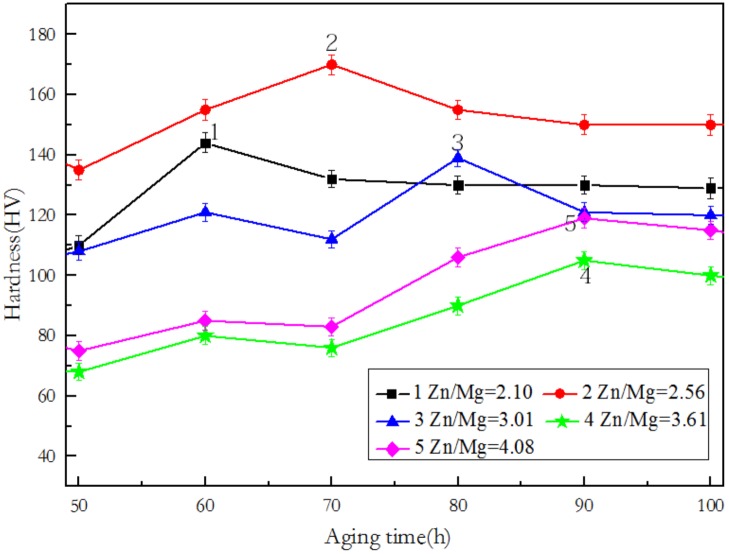
The age-hardness curves of the alloys during isothermal aging at 120 °C.

The age-hardness curves of the alloys during isothermal aging at 120 °C are shown in Figure 1. As shown in Figure 1, we can see that with the increase in Zn/Mg ratio, the time at which the alloy reaches the peak hardness gradually increases. Alloy 2 with Zn/Mg ratio of 2.56 obtains the highest hardness value and the peak hardness of Alloy 4 with Zn/Mg ratio of 3.61 is the lowest according to the hardness curves. As such, there is no direct relation between the peak hardness and the ratio of Zn/Mg. Comparing the hardness curves of Alloy 1 and Alloy 2, it can be found that when the content of Mg is approximately equal, with the increase in Zn content, the hardness value and the time to reach the peak hardness both increases. While the Mg contents of Alloy 4 and Alloy 5 are also approximately equal, the increase in Zn content does not delay the time for the alloy to reach the peak hardness. This indicates that when Zn/Mg is relatively low, the increase in Zn content delays the time when the alloy reaches the peak hardness point. The Zn content of Alloy 1 and Alloy 4 is approximately equal, the Mg content is quite different, and the peak hardness and the time reaching peak hardness are greatly different. The effects also can be seen by comparing the hardness curves of Alloy 2 and Alloy 5.This indicates that the change in Mg content controls the peak hardness of the alloy and the time to reach the peak value. When the Zn/Mg ratio is greater than 3.01, two aged peak were observed, which is similar to the study of other 7xxx aluminum alloy. The results of the literatures [25,26] show that the first peak is due to GP II and the second peak results from η’ phase. The description of those two phases is given in the discussion section.

**Figure 2:**
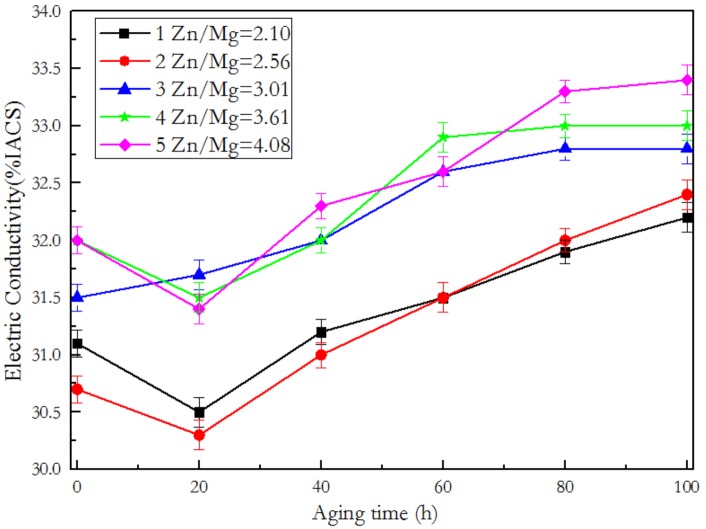
The conductivity curves of the alloys during isothermal aging at 120 °C.

Figure 2 shows the conductivity curves of the alloys during isothermal aging at 120 °C. When the alloys were casted, deformed, and compressed under the same conditions, the conductivity values after quenching is mainly determined by the supersaturated solid solubility of solution elements such as Zn and Mg within Al matrix. When the solution temperature of 460 °C or higher, elements such as Zn and Mg can be completely dissolved in Al matrix and form a supersaturated solid solution with Al matrix in the composition range of 7005 alloy. From the test components in Table 1, it is known that only the contents of Zn and Mg are changed in the ratio of five alloys, so the electrical conductivity at quenched state is only related to the total contents of Zn and Mg. Comparing the electrical conductivity value of five alloy at peak-aged point, it can be found that as the Zn/Mg ratio increases, the electrical conductivity value also increases. Therefore the Zn/Mg ratios have corresponding relation with the electrical conductivity values of the peak-aged hardness point of the alloys.

### 3.2. Precipitation Behaviors in Relation with the Zn/Mg Ratio of the Alloy

**Figure 3:**
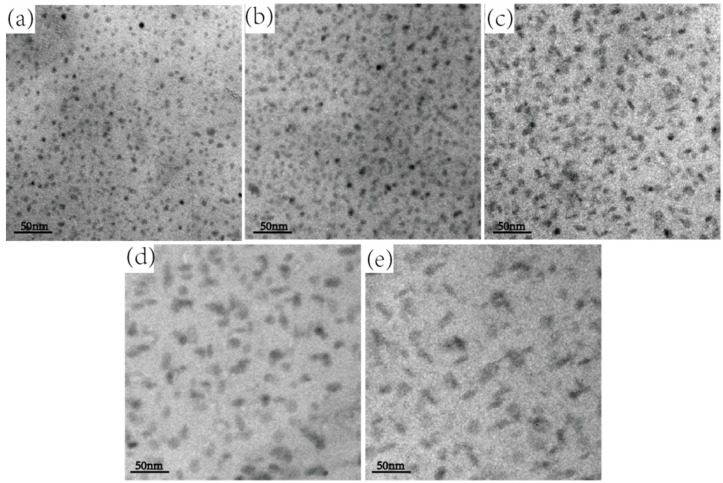
The intragranular precipitate phase of the alloys (in <001>_Al_ zones axis): (**a**) Alloy 1, aging 61 h; (**b**) Alloy 2, aging 69 h; (**c**) Alloy 3, aging 81 h; (**d**) Alloy 4, aging 89 h; (**e**) Alloy 5, aging 90 h.

Figure 3 shows the TEM bright field images of the intragranular precipitate phase at the peak-aged hardness state of five alloys. Comparing the intragranular precipitation phases of five alloys, it can be found that as the Zn/Mg ratio increases, the number of intragranular precipitate phase gradually decreases and the size gradually increases. Specifically, the intragranular precipitate phase of Alloy 1 is the smallest and most dense and the precipitate phases are mostly in the form of small discs; the size of the intragranular precipitate phase of Alloy 2 is slightly larger than that of Alloy 1, most of which is in the form of disk, and a small portion is in the form of short rod; the precipitate phase of Alloy 3 exhibits short rod shape; the precipitate phase of Alloy 4 is mainly appear as clear short rod shape and a small number of discs; the precipitate phase of Alloy 5 is mainly short rod-shaped and its size is the largest.

**Figure 4:**
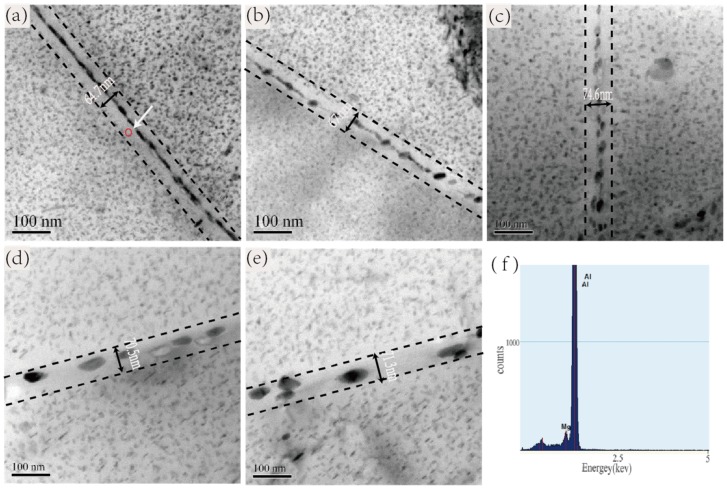
Grain boundary precipitation phase of the alloys: (**a**) Alloy 1, aging 61 h; (**b**) Alloy 2, aging 69 h; (**c**) Alloy 3, aging 81 h; (**d**) Alloy 4, aging 89 h; (**e**) Alloy 5, aging 90 h; (**f**) the energy spectrum at the dotted line of Alloy 1.

Figure 4 is the TEM bright field images of the grain boundary at the peak-aged hardness state of five alloys and an energy spectrum of the microdomain near the grain boundary of Alloy 1. It can be observed that as the Zn/Mg ratio increases, the grain boundary precipitate phase of alloy gradually becomes larger, and gradually changes from a continuous distribution state to a discontinuous distribution state, moreover the width of PFZ gradually increases and then gradually remains unchanged. Comparing the PFZ width of the grain boundary in Figure 4, it can be found that the width of PFZ does not change much and the value is between 64 and 74 nm, however the size variety of the precipitate phase in grain boundary is obvious. 

### 3.3. Mechanical Properties and Resistance of Stress Corrosion Cracking in Relation with the Zn/Mg Ratio of the Alloy 

In order to calculate the size and distribution of precipitate phase after peak-aged, 20 crystal grains were randomly selected to characterize in <001> zones axis, as shown in Table 2. Since the precipitate phases of the 7xxx alloy is very small, the average of the area fraction “M” is used to represent the proportion of the precipitate phases within the Al matrix, and the calculation formula is:

(1) $$M=\frac{1}{n}\sum_{1}^{n}\frac{\int_{0}^{\sqrt{L}}fn}{L}$$

L is the unit area, and the integral of *f*(*n*) is the area of the precipitate phase in the nth unit area.

According to the study by Starink, M.J. [27,28] et al., the yield strength of the 7xxx aluminum alloy can be calculated by the following formula:

σ_y_ = △σ_gb_ + M△τ_(tot)_= △σ_gb_ + M (△τ_int_ + △τ_ss_ + △τ_p_ + △τ_d_). (2)

In the formula, △σ_gb_ is the contribution of grain boundary strengthening to the yield strength, M is the Taylor index, △τ_int_ is the critical shearing stress (CRSS) of pure aluminum, and △τ_ss_, △τ_p_, △τ_d_ are the contribution of intragranular dislocations, solid solution strengthening, and precipitation strengthening to CRSS respectively.

For aging-strengthened aluminum alloys, precipitate strengthening contributes most to the strength of alloy and the strengthening level is mainly determined by the volume fraction, size and distribution of precipitate phase. If the number of precipitated phases is larger and its size is smaller, the increment of Δτ_p_ will be more. According to Table 3, with the increase in Zn/Mg ratio, the yield strength of alloy in the air increases first and then decreases and the elongation gradually increases. The matrix precipitate (MPs) and the grain boundary precipitate phase (GPBs) of Alloy 1 and Alloy 2 have both a higher unit area fraction and smaller size in Table 2, so the yield strength of the two alloys in the air is relatively higher. On the other hand the precipitate phase of Alloys 4 and 5 are larger in size and have a low fraction per unit area, so their yield strength is lower.

The precipitate phases of the 7xxx aluminum alloy in peak-aged state are strictly coherent or semi-coherent with Al matrix. When deformation occurs, the dislocation lines pass through the second phase with the cut-off form. Since the number of precipitate phase within the matrix is much higher than that of the grain boundary, the dislocation lines are mostly entangled within the crystal. The greater the number of precipitate phases in the crystal, the greater the number of dislocation lines that are entangled in it, the smaller the ability of alloy to accommodate dislocation [29,30,31,32]. The ability to accommodate dislocation can indirectly reflect the deformation properties. It can be seen from Table 2 that the intragranular precipitate phase of Alloy 1 and Alloy 2 are small in size and densely distributed, and these precipitate phases are mostly in coherent and semi-coherent relation with the matrix [14,15], thus the elongation of Alloy 1 and Alloy 2 are lower. In contrast the intragranular precipitate phases of Alloy 4 and Alloy 5 are larger in size and distributed in dispersion, and thus have higher elongation.

Table 3 shows the results of the SSRT test of alloy in the air and 3.5% NaCl + 0.5% H_2_O_2_ aqueous solution respectively after peak-aged. Under the corrosive environment of 3.5% NaCl + 0.5% H_2_O_2_ aqueous solution, the resistance of stress corrosion cracking of alloy gradually increased with the increase in Zn/Mg ratio. These results indicate that there is a correspondence relation between the Zn/Mg ratio and the resistance of stress corrosion cracking of alloy at peak-aged state.

## 4. Discussion

### 4.1. Effect of Zn/Mg Ratio on Peak Hardness, Aging Time, and Conductivity

As shown in Figure 1 and Table 3, the order of the hardness and strength of the alloy from high to low is: Alloy 2, Alloy 1, Alloy 3, Alloy 5, and Alloy 4. There is no direct relation with respect to the hardness, strength and Zn/Mg ratio of the alloy. As shown in Figure 3 and Figure 4, it can be found that the microstructure of Alloy 1 and Alloy 2 (Zn/Mg ratio ≤ 2.62) are very similar, and their precipitate phases are mostly like the shape with small disc. Meanwhile the precipitate phases within grain boundary are fine and continuous, and their PFZ are relatively narrow. In addition they show the same changing trend in the hardness curve and the conductivity curve with aging time. Alloy 4 and Alloy 5 (Zn/Mg ratio ≥ 3.61) also show the similar outcomes of microstructure and mechanical properties according to Figure 3 and Figure 4. This indicates that within the composition range of 7005 aluminum alloy, the Zn/Mg ratio has a clear decisive effect on the type, size and distribution of the precipitate phase during the aging at 120 °C.

**Figure 5:**
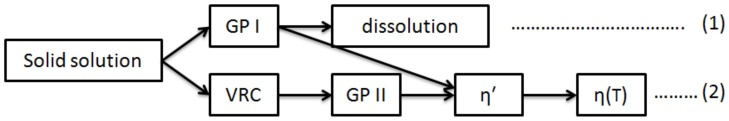
The aging precipitation sequence of 7xxx aluminum alloys.

**Figure 6:**
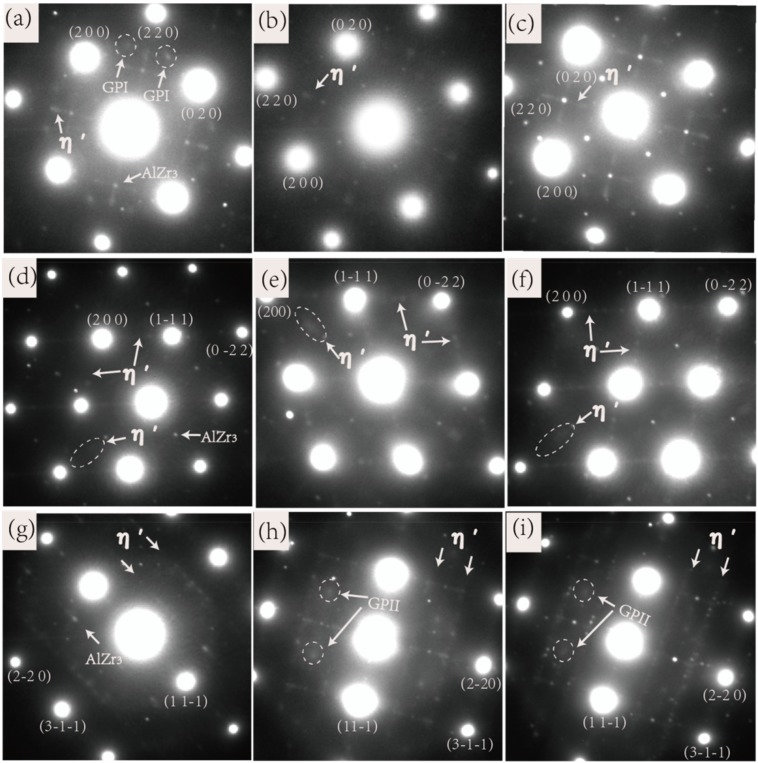
SAD (selected area diffraction) patterns of Alloy 1 aged for 61 h at 120 °C: (**a**) <001>_Al_, (**d**) <110>_Al_, (**g**) <112>_Al_ respectively; SAD patterns of Alloy 3 aged for 81 h at 120 °C: (**b**) <001>_Al_, (**e**) <110>_Al_, (**h**) <112>_Al_ respectively; SAD patterns of Alloy 5 aged for 90 h at 120 °C: (**c**) <001>_Al_, (**f**) <110>_Al_, (**i**) <112>_Al_ respectively.

Both the electrical conductivity of the peak-aged state and the aging time taken for reaching peak hardness have a good correspondence relation with the Zn/Mg ratio. That means the higher the Zn/Mg ratio, the higher the conductivity of the alloy in the peak-aged state and the longer it takes to reach the peak-aged state. The results are mainly due to the difference of the nucleation activation energy of the precipitate phase controlled by the Zn/Mg ratios. The precipitate sequence of the 7xxx aluminum alloy is: α solid solution → GP zones → η′ phase → η phase (T phase), where in the GP zones are further divided into the GP I zones and the GP II zones according to different formation conditions, as shown in Figure 5. Figure 5 shows the aging precipitation sequence of 7xxx aluminum alloys in two different ways. Path (1) indicates that the Mg and Zn atoms after solid solution can directly form GP I and then transform into the η’ phase or dissolve into MgZn cluster with too long aging time, while the path (2) indicates that VRC (vacancy-rich clusters) should be formed before the formation of GP II, and finally GP II can be converted into metastable η’ phase. Obviously, the formation of GP II is more complex and difficult than GP I. The nucleation conditions of the GP II zones [14,15,21] are that the solution temperature must be greater than 450 °C, the quenching temperature is not higher than 40 °C, and the aging temperature is higher than 70 °C. The nucleation conditions of the GP I zones are less rigorous, and the nucleation temperature ranges from 25 °C to 140 °C, and the conditions are independent of the solution temperature and quenching temperature. According to the research in the literatures [14,15,21], the optimal crystal band axis to observe the GP I zones, GP II zones and η’ phase are <001>, <112>, and <110> zones axis of Al matrix respectively. Figure 6 is the diffraction pattern related to the peak-aged of the alloys, Figure 6a,d,g are in <001>_Al_ zones axis, Figure 6b,e,h are in <110>_Al_ zones axis, and Figure 6c,f,i are in <112>_Al_ zones axis respectively. Figure 6a is the diffraction spot of the η’ phase and the GP I zones under <001>_Al_ zones axis, and no diffraction spot of the GP II is observed in Figure 6g, indicating that Alloy 1 with lower Zn/Mg ratio mainly nucleates as the GP I zones with the lower nucleation energy, and then begins to transform to η′ phase. In addition, the GP I zones and the η′ phase are both the main hardening and strengthening phase of the 7xxx aluminum alloy. Therefore Alloy 1 reached the peak hardness value up to 60 h. Alloy 2 also has the same diffraction pattern. Figure 6h,i are TEM diffraction spots of Alloy 3 and Alloy 5 respectively, and it is known from the literatures [14,21] that the dominating precipitate phase are the η’ phase and the GP II zones. The GP II zones nucleate and grow on the atomic planes filling with supersaturated vacancies and Zn-rich or Al-Zn-rich atoms after quenching. Therefore, the aluminum alloy with high Zn/Mg ratio will form more GP II zones in the early aging stage, and the nucleation energy of the GP II transferring to the η’ phase is much higher than that of the GP I zones to the η’ phase, and thus longer time to transform into the η’ phase. That is the reason why the aging time of Alloy 3, Alloy 4, and Alloy 5 reaching the peak hardness point gradually become longer.

The change in conductivity of the alloy at the peak hardness point is also due to the nucleation energy of the precipitate phase resulting from the different Zn/Mg ratio. The GP zones of Alloy 1 has lower nucleation energy, so it is easy to nucleate and transfer, in a shorter time, to the η’ phase with small size. As shown in Figure 6d, the η’ phase diffraction spot of Alloy 1 (the ellipse and the arrow in the figure are marked) are dark, and no obvious bright streaks appear, which indicates that the η’ phase is in the initial stage that the η’ phase transfer to the η phase. At this time, most of the η’ phase and the Al matrix are in a strict coherent and semi-coherent crystal orientation relationship, so the electrical conductivity of the alloy is low. However, the η′ phase diffraction spots of Alloy 3 and Alloy 5 (Figure 6e,f) are much brighter and have multiple orientations with the matrix, which indicates that the η’ phase is in the mid-late phase of the transformation to the η phase [33]. The η’ phase at this stage are semi-coherent and non-coherent with the Al matrix, and therefore the electrical conductivity of the alloy is higher.

### 4.2. Influence of Zn/Mg Ratio on SCC Performance

According to the previous discussion of the relationship between the Zn/Mg ratio and the electrical conductivity of the alloy, it is known that the Zn/Mg ratio corresponds to the electrical conductivity at the peak hardness state. The electrical conductivity generally corresponds with the resistance of stress corrosion cracking of the alloy, indicating that the alloy with high conductivity generally owns good resistance of stress corrosion cracking [8,34]. Alloy 1 is composed of the GP I zones and the η’ phase with small size. The chemical composition of the GP I zones has not yet been determined. The research on the 7050 alloy in the literatures [7,20,35,36] shows that the GP I zones appears in the early aging stage and are generally small MgZn clusters, which can convert into the η’ phase and the η phase in the later aging stage. However, those GP I with smaller size would dissolve during the aging process forming free Mg and Zn atoms within the matrix, as the path (1) shown in Figure 5. It is easier for the smaller MgZn clusters formed during the peak-aged stage to decompose in the Cl^−^ and H^+^ environments, and form Mg-H corrosion channels with the H elements, which would accelerate the expansion of micro cracks. As shown in Figure 4f, the energy spectrum analysis of the region near the grain boundary reveals that certain amount of Mg element distributes along the boundary. As such, the grain boundary and those area near to them are in Mg-rich state, which is easier to occur Mg-H effect and form an accelerated cracking corrosion crack. The results in Table 2 also statistically indicate that the grain boundary precipitate phase of Alloy 1 is slender and continuous. According to the anodic dissolution mechanism, the grain boundary precipitate phases are more negative than the Al matrix, and acts as an anode in the galvanic cell formed in the Cl^−^ + H^+^ aqueous solution. The precipitated phase which are small in size and continuously distributed on the grain boundaries, tend to form a “paralleling battery” with Al matrix, which will accelerate the dissolution of the precipitated phases. Thus it is easy for such area to become a corrosion channel and a source of the growth of stress corrosion cracking.

The precipitate phases at the peak-aged state of Alloy 4 and Alloy 5 are mainly η’ phase with larger size and GP II zones. The stability of the GP II zones are higher than that of the GP I zones, so it is relatively difficult for the GP II zones to dissolve in the Cl^−^ and H^+^ aqueous solution and difficult to form Mg-H corrosion channel with the H element. In addition from the statistical results of Table 2, it is known that the intragranular precipitate phase of Alloy 4 and Alloy 5 are mostly the η’ phases with short rod-shaped size, and the average size of the precipitate phase within the grain boundary is larger than other alloys. Moreover, they exhibit intermittent distribution and their PFZ are narrower. According to the literature [37], it is not easy of such microstructure to form corrosion channels, so they have better resistance to stress corrosion cracking.

**Figure 7:**
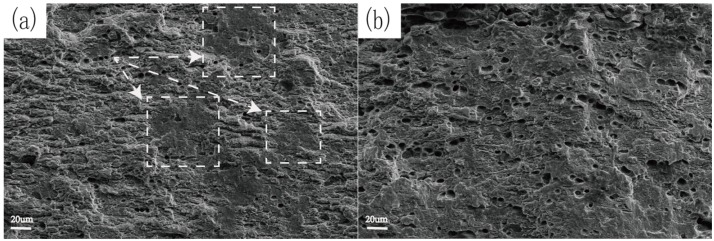
Fracture morphologies for Alloy 1 (aged for 61 h at 120 °C) and Alloy 5 (aged for 90 h at 120 °C) in 3.5% NaCl + 0.5% H_2_O_2_ aqueous solution.

Figure 7 shows the SEM morphology of intermediate fracture surface of Alloy 1 and Alloy 5 at peak aging state after immersed in 3.5% NaCl + 0.5% H_2_O_2_ aqueous solution. As shown in the boxed area of Figure 7a, the fracture of Alloy 1 shows more cleavage layers. This indicates that the fracture type of Alloy 1 was a combination of transgranular and intergranular characteristics accompanied with some local erosion. It also can be seen from Figure 7b that there were more transgranular cracks in Alloy 5. This directly proves that the Alloy 5 at peak aging state has better resistance of stress corrosion cracking than Alloy 1 in 3.5% NaCl + 0.5% H_2_O_2_ aqueous solution.

## 5. Conclusion

In the aqueous solution of Cl^−^ + H^+^, it is easier for GP I zones and the η’ phase with smaller size to dissolve than GP II and the η’ phase with larger size. This difference causes the degree of difficulty in the occurrence of the Mg-H effect to be different. The difference in the size and distribution of the precipitate phase within the grain boundary results in different rates of electrochemical corrosion. Therefore the Zn/Mg ratio affects the resistance of stress corrosion cracking of the peak-aged alloy by controlling the type, size, and distribution of the precipitate phase. Through the study of the 7005 aluminum alloy in this paper, we can draw the following conclusions:

1. As the Zn/Mg ratio increases, the aging time to reach the peak-aged hardness gradually increases. When the Zn/Mg ≤ 2.6, the intragranular precipitate phases mainly consist of GP I zones and the disc-like η’ phase with small size. The grain boundary precipitates are slender and continuous, and the alloy has poor resistance of stress corrosion cracking in 3.5% NaCl + 0.5% H_2_O_2_ aqueous solution.
2. When the Zn/Mg ≥ 3.61, the intragranular precipitate phase of alloy are mainly composed of GP II zones and the short-rod η’ phase with larger size. The grain boundary precipitates are larger and intermittently distributed and the PFZ is narrow, which means the alloy has good resistance to stress corrosion cracking in 3.5% NaCl + 0.5% H_2_O_2_ aqueous solution.

## Figures and Tables

**Table 1:** Test compositions of the examined alloys (wt. %).

No.	Zn	Mg	Mn	Cu	Cr	Zr	Al	Zn/Mg
1	4.201	1.850	0.256	0.099	0.148	0.145	93.301	2.27
2	4.953	1.890	0.254	0.097	0.149	0.145	92.512	2.62
3	4.556	1.512	0.254	0.099	0.146	0.146	93.287	3.01
4	4.149	1.149	0.256	0.098	0.148	0.145	94.055	3.61
5	4.905	1.202	0.251	0.097	0.145	0.145	93.255	4.08

**Table 2:** Statistical results of precipitate phases (in <001>_Al_ zones axis).

No.	Rod Length of MPs (nm)	Disk Diameter of MPs (nm)	Average Area Fraction of MPs (%)	Average Length of GBPs (nm)	Average Width of GBPs (nm)	Average Width of PFZ (nm)
1	5.7–17.5	3.5–18.5	41.2 ± 2.4	80.3 ± 9.5	15.36 ± 5.5	62.22
2	5.5–25.2	5.3–30.9	45.8 ± 2.2	30.3 ± 13.2	25.55 ± 7.1	65.08
3	11.4–30.2	10.6–45.5	33.6 ± 2.2	31.4 ± 10.8	35.8 ± 10.1	72.26
4	18.9–45.1	17.3–55.1	25.1 ± 2.2	59.8 ± 16.9	62.2 ± 20.5	69.33
5	32.8–65.8	22.2–65.7	27.5 ± 2.3	64.4 ± 21.4	70.5 ± 16.5	70.57

MPs (the grain boundary precipitate phase); GBPs (the grain boundary precipitate phase); PFZ (the precipitation free zone).

**Table 3:** The results of SSRT tests (strain rate 8.34 × 10^−7^ s^−1^).

No.	σ_y_(air) (MPa)	σ_y_(NaCl + H_2_O_2_) (MPa)	∆(air) (%)	∆(NaCl + H_2_O_2_) (%)	Rscc
1	451	377	13.33	3.1	0.835
2	503	426	13.74	3.24	0.846
3	422	381	15.66	3.86	0.906
4	311	288	17.09	4.25	0.925
5	336	314	17.11	4.47	0.936

σ_y_ indicates the yield strength and the symbol of “∆” indicates elongation.
